# Nanometer-thick Si/Al gradient materials for spin torque generation

**DOI:** 10.1126/sciadv.adr9481

**Published:** 2025-05-09

**Authors:** Taisuke Horaguchi, Cong He, Zhenchao Wen, Hayato Nakayama, Tadakatsu Ohkubo, Seiji Mitani, Hiroaki Sukegawa, Junji Fujimoto, Kazuto Yamanoi, Mamoru Matsuo, Yukio Nozaki

**Affiliations:** ^1^ Department of Physics, Keio University, Yokohama 223-8522, Japan.; ^2^ Research Center for Magnetic and Spintronic Materials, National Institute for Materials Science, 1-2-1 Sengen, Tsukuba 305-0047, Ibaraki, Japan.; ^3^ Graduate School of Science and Technology, University of Tsukuba, Tsukuba 305-8577, Japan.; ^4^ Kavli Institute for Theoretical Sciences, University of Chinese Academy of Sciences, No. 3, Nanyitiao, Zhongguancun, Haidian District, Beijing, China.; ^5^ CAS Center for Excellence in Topological Quantum Computation, University of Chinese Academy of Sciences, Beijing 100190, China.; ^6^ RIKEN Center for Emergent Matter Science (CEMS), Wako, Saitama 351-0198, Japan.; ^7^ Advanced Science Research Center, Japan Atomic Energy Agency, Tokai 319-1195, Japan.; ^8^ Center for Spintronics Research Network, Keio University, Yokohama 223-8522, Japan.

## Abstract

Green materials for efficient charge-to-spin conversion are desired for common spintronic applications. Recent studies have documented the efficient generation of spin torque using spin-orbit interactions (SOIs); however, SOI use relies on the employment of rare metals such as platinum. Here, we demonstrate that a nanometer-thick gradient from silicon to aluminum, which consists of readily available elements from earth resources, can produce a spin torque as large as that of platinum despite the weak SOI of these compositions. The spin torque efficiency can be improved by decreasing the thickness of the gradient, while a sharp interface was not found to increase the spin torque. Moreover, the electric conductivity of the gradient material can be up to twice as large as that of platinum, which provides a way to reduce Joule heating losses in spintronic devices.

## Teaser

Silicon-aluminum compositional gradients enable efficient charge-to-spin conversion comparable to platinum.

## INTRODUCTION

Nonequilibrium spin polarization in conduction electrons enables the magnetization direction to be manipulated via spin torque using spin-orbit interactions (SOIs) in spintronic devices such as magnetic random-access memory (*1*, *2*) and spin torque nanooscillators (*3*–*5*). Phenomena such as the spin Hall effect (SHE) (*6*–*10*) and the Rashba-Edelstein effect (REE) (*11*–*14*) are commonly used to generate spin-polarized flow, i.e., spin currents (SCs), in strong SOI materials. Such nonequilibrium spin polarization generally requires specific materials with a strong SOI, usually consisting of heavy 5d metal elements such as tantalum (Ta), tungsten (W), and platinum (Pt). The figure of merit (FOM) for SC generation capability is generally determined as the product of the spin Hall angle and the electric conductivity of the material. Namely, higher-conductivity materials exhibit superior SC generation, while stronger-SOI elements generally exhibit smaller conductivities (*15*–*19*). The material choice dilemma for SC generation creates a bottleneck in spintronic device production. Poor conductivity in SC circuits can lead to other serious problems such as wiring delays and Joule losses in integrated circuits. Moreover, semiconductor device performance is often degraded by contamination with strong-SOI elements. Therefore, a SOI-free technology is important for enabling SC circuit integration in electric devices. The problem of rare-metal element scarcity also poses a serious challenge to sustainable development.

**Fig. 1.**
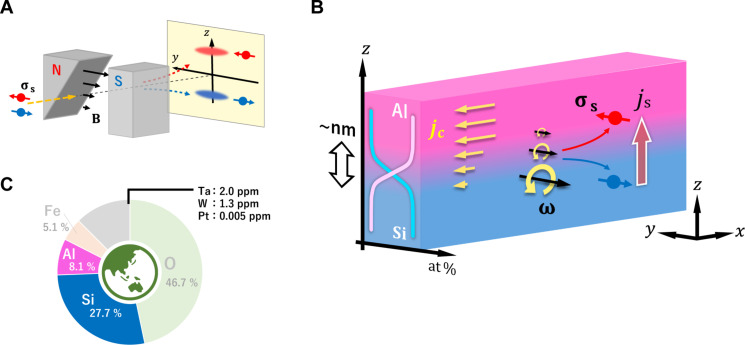
Green and rare metal–free composition gradient material for spin current generation. (**A**) Schematic illustration of the Stern-Gerlach experiment. Quantization of spin angular momentum σ_**s**_ was observed when a beam of silver atoms was split in two as it passed through a gradient of a static magnetic field **B**. (**B**) Schematic image of a fabricated Si/Al gradient material. The Cartesian coordinate system used throughout this article is also shown in (B). A nanometer-thick compositional gradient from Si to Al exists along the *z* axis with much more conductivity in the Al layer than the Si layer. When an electric current *j*_c_ is applied along the *x* axis, an electric current vorticity ω = **∇** × ***j***_**c**_ appears, whose vector points along the *y* axis. The electron spin σ_s_ in the gradient material is polarized along the *y* axis by an emergent magnetic field because of the vortical electric current. As a consequence, the gradient of ω leads to a density gradient in the nonequivalent electron spin, which produces a spin current along the *z* axis. (**C**) Plot of relative elemental abundance in Earth’s crust. Si and Al represent the most abundant semiconductor and metal materials, respectively, while typical large-spin-current source materials such as Ta, W, and Pt are very rare.

A promising technology for producing flow of spin angular momentum without using SOI is spin separation produced by a magnetic field gradient. Stern and Gerlach (*20*, *21*) demonstrated spin separation in 1922 using a field gradient (*z* axis in Fig. 1A). Although their approach relies on a magnetic field gradient, it established an important precedent for exploring how spatially varying field can affect spins, even without strong SOI. Subsequently, Matsuo *et al.* (*22*) introduced the concept of spin-vorticity coupling (SVC), where the macroscopic vorticity ω and spins **s** interact via the Hamiltonian H∝−s·ω. Namely, ω acts as an effective magnetic field. Notably, while the original Stern-Gerlach experiment dealt with a single particle, the spin-vorticity concept extends this idea to nonequilibrium many-body systems. In this picture, a nonuniform vorticity (∇ω ≠ **0**) leads to a nonequilibrium spin state, giving rise to a spin current. Experiments on metallic liquid (*23*), thin copper (Cu) films (*24*–*26*), and quark-gluon plasma (*27*, *28*) have demonstrated that effective magnetic fields can arise from this gyromagnetic effect. A related phenomenon in surface- and edge-oxidized Cu films (*29*, *30*) further supports the idea that a nonuniform electric current can generate an effective magnetic field via SVC. Because a conductivity gradient induces vorticity in the electric current (ω = ∇ × *j*_c_), this magnitude should depend on how abruptly the electron conductivity transitions within an oxide (Fig. 1B). Our focus thus centers on leveraging such gradients to produce spin currents independently of heavy-element SOI, expanding the range of materials available for spintronic applications. However, the thickness of the oxidation gradient is difficult to control atomically. This poses a problem not only for practical applications of spintronic devices but also for confirming the existence of emergent magnetic fields because of electric current vorticity.

In this study, we successfully fabricated a nanometer-thick artificial gradient from silicon (Si) to aluminum (Al), which constitutes the second- and third-most abundant components in Earth’s crust (Fig. 1C), and we demonstrate its ability to generate a spin torque comparable to Pt. The magnitude of the spin torque increases when the compositional gradient steepens; however, an atomically sharp interface does not substantially increase the spin torque. Moreover, as reported in the study of a surface-oxidized Cu film (*29*), we observed a large nonreciprocity that suggests the SC generation via an emergent magnetic field because of electric current vorticity.

## RESULTS

### Fabrication and structural analysis of Si/Al gradient materials

**Fig. 2.**
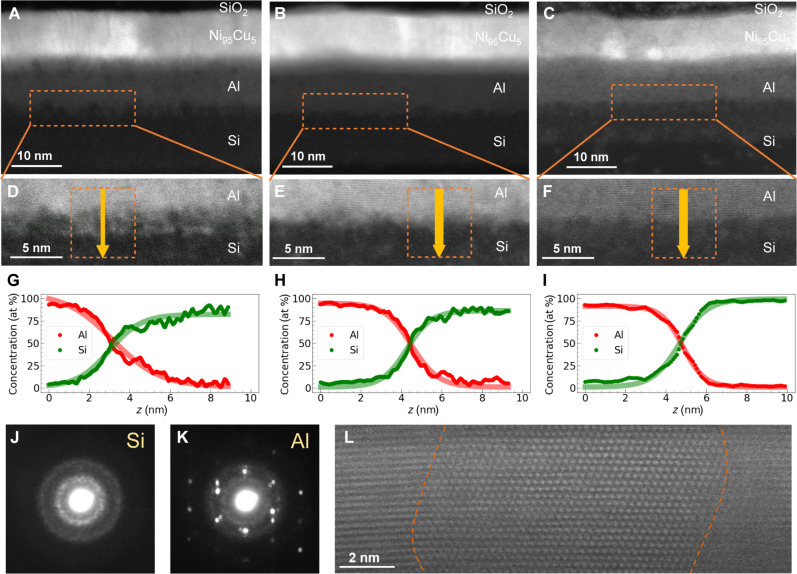
Microstructural and compositional characterization of gradients across the Si/Al interface. (**A** to **C**) HAADF-STEM images of samples with (A) *t*_i_ = 2.0 nm, (B) 1.0 nm, and (C) 0.0 nm. (**D** to **F**) Enlarged views of the sample near the area analyzed by EDS. EDS profiles were generated from within the orange dashed boxes. (**G** to **I**) Averaged profiles of the atomic concentration of Si and Al for each sample along the arrows in each box. (**J** and **K**) Nanobeam diffraction patterns for Si and Al layers, respectively. (**L**) High-resolution HAADF-STEM image of the Al layer. Dashed lines indicate the grain boundaries.

We fabricated Si(10)/Al(*t*_i_/2)/Si(*t*_i_/2)/Al(10)/Ni_95_Cu_5_(10)/SiO_2_(20) (unit: nm) multilayer strips on a thermally oxidized Si substrate by means of a conventional liftoff method using magnetron sputtering and photolithography. Here, *t*_i_ is the thickness of the interfacial Al/Si insertion, which varied from 0.25 to 2.0 nm at intervals of 0.25 nm. Al is a very conductive metal with an open 2p shell. Although Si is adjacent to Al in the periodic table, it is a semiconductor with much lower electrical conductivity than Al. Sputter deposition processes generally lead to atomic or metallographic disturbances at an interface because of the large kinetic energy of the sputtered particles. An insertion of a few nanometers of Al/Si therefore increases the mixed region at the interface between 10-nm-thick Si and Al layers. Microstructural analysis using high-angle annular dark-field scanning transmission electron microscopy (HAADF-STEM) revealed an increase in the thickness of the compositional gradient from Si to Al with increasing *t*_i_. Figure 2 (A and B) displays cross-sectional HAADF-STEM images near the Si/Al interface with *t*_i_ = 2.0 and 1.0 nm, respectively. In comparison, a cross section of a Si/Al interface without Al/Si insertion (i.e., *t*_i_ = 0 nm) is also shown in Fig. 2C, in which a sharp interface was clearly observed. The Al/Si insertion obscures the Si/Al boundary. Moreover, aggregation of Al and/or Si was observed at the Si/Al interface for *t*_i_ = 2.0 nm. The formation of such aggregation features is attributed to nonsolid solution atomic mixing between Si and Al. Energy-dispersive spectroscopy (EDS) line profiles of each element (Si and Al) were obtained by averaging the signals in the dashed box area shown in Fig. 2 (D to F), which explicitly confirm the formation of a Si/Al gradient with a transition thickness that systematically varies with *t*_i_. The transparent bold lines in Fig. 2 (G to I) show the best fit to the following equation

$$\text{Composition}=\frac{C_{1}+C_{2}}{2}\pm \frac{C_{2}-C_{1}}{2}\text{tanh}\left(\frac{z-z_{\text{int}}}{L}\right)$$ (1)

where *C*_1_ and *C*_2_ are the composition at each end of an EDS scan, *z*_int_ is the center position of the interface, and *L* is the thickness of the compositional gradient from Si to Al. The fit of Eq. 1 indicates *L* values of 1.3 and 2.4 nm for *t*_i_ = 1.0 and 2.0 nm, respectively. As shown in Fig. 2, some compositional fluctuations occur along the interfaces, especially for the sample with *t*_i_ = 2.0 nm; thus, the value of *L* depends on both the size and position of the EDS signal-averaging window. The value of *L* determined in Fig. 2 should be regarded as an average thickness of the compositional gradient. It is known that Al and Si form a typical nonsolid solution combination at equilibrium. Few intermetallic compound or intermediate phases of these elements exist, although certain nonequilibrium states can form during the sputter deposition process at room temperature. Figure 2 (J and K) shows nanobeam electron diffraction patterns for 10-nm-thick Si and Al layers, respectively. A broad halo pattern from the amorphous structure of the Si layer is present, whereas diffraction spots corresponding to the polycrystalline nature of the Al layer appear in the high-resolution HAADF-STEM image in Fig. 2L.

### Electric current–induced spin torque

When an electric current is applied to the bilayer strip consisting of a nonmagnet (NM) and a ferromagnet (FM), part of the SC generated in the NM is transmitted toward the FM followed by a spin torque applied on the magnetization of the FM. This torque is generally referred to as a damping-like (DL) torque, τ_DL_, whereas a separate spin torque known as the field-like (FL) torque, τ_FL_, arises from the SC scattered at the NM/FM interface. The effect of these torques on a magnetization **m** produced by an SC with a polarization σ_**s**_ is described by the following equation

$$\begin{array}{c} \tau =\tau_{\text{DL}}+\tau_{\text{FL}}\\ =\xi_{\text{DL}}j_{c}\frac{\hbar }{2e}\frac{1}{\mu_{0}M_{s}d_{\text{FM}}}m\times \left(m\times \sigma_{s}\right)+\xi_{\text{FL}}j_{c}\frac{\hbar }{2e}\frac{1}{\mu_{0}M_{s}d_{\text{FM}}}m\times \sigma_{s} \end{array}$$ (2)

where ξ_DL_ and ξ_FL_ are the efficiencies of the DL and FL torques with respect to an electric current density *j*_c_, while *e*, μ_0_, and *ℏ* are the elementary charge, permeability of a vacuum, and the reduced Planck’s constant, respectively. *M*_s_ and *d*_FM_ are the saturation magnetization and FM thickness, respectively. Both these orthogonal torques play an important role in magnetization switching, which has been widely investigated in applications for nonvolatile magnetoresistive memory and magnetic logic devices.

**Fig. 3.**
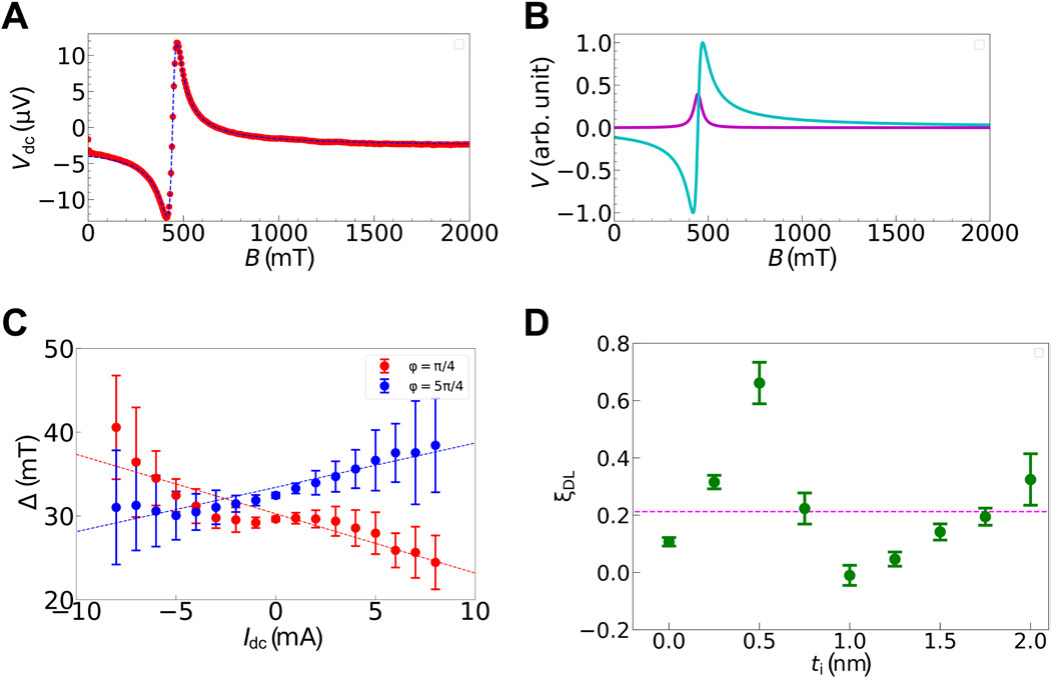
ST-FMR experiment for evaluating electric current–induced spin torque. (**A**) ST-FMR spectrum measured for a Si/Al/Ni_95_Cu_5_ trilayer film with *t*_i_ = 0.5 nm at a microwave frequency of 20 GHz. The dashed curve represents the best-fit result for a combination of symmetric and antisymmetric Lorentzian functions. (**B**) Symmetric (purple) and antisymmetric (blue) Lorentzian components included in (A). (**C**) FMR linewidth Δ as a function of dc current *I*_dc_ for a sample with *t*_i_ = 0.5 nm. The red and blue closed circles represent the results measured for a positive (φ = π/4) bias magnetic field and a negative (φ = 5π/4) bias magnetic field. Dashed lines indicate the results of linear fit. The vertical error bars indicate the standard deviation of 10 times measurements. (**D**) Damping-like torque efficiency ξ_DL_ as a function of *t*_i_. The vertical bars indicate the standard deviation of 20 times measurement. The closed circles indicate the ξ_DL_ values for samples with *t*_i_ ranging from 0 to 2.0 nm. The dashed line shows the ξ_DL_ value measured for the Pt(10)/Ni_95_Cu_5_(10) bilayer.

To evaluate the strength of the DL torque produced by applying an electric current to the sample, we conducted direct current (dc) Gilbert damping modulation of spin torque ferromagnetic resonance (ST-FMR) spectrum (*15*, *16*, *18*, *31*–*34*). The theory of this technique is described in Supplementary Text S1. In the ST-FMR experiment, an alternating current was applied to the strip using a microwave with an amplitude of 20 dBm and a frequency of 20 GHz. The dc voltage *V*_dc_ because of the ST-FMR excitation was measured while sweeping the external field from 0 to 2.0 T. The measured spectrum in the absence of a dc is shown in Fig. 3A for *t*_i_ = 0.5 nm, which can be reproduced by a combination of symmetric and antisymmetric Lorentzian functions, as shown in Fig. 3B. When a dc *I*_dc_ is applied simultaneously, a DL torque produced by the dc modulates the Gilbert damping of ferromagnetic resonance (*15*, *32*). As shown in eq. S6, the linewidth of the ST-FMR spectrum, Δ, was changed as a function of *I*_dc_. Last, we can evaluate ξ_DL_ from the slope of the linear relation between Δ and *I*_dc_. It should be noted that another spin torque, i.e., the FL torque, does not contribute to the change in the linewidth at all.

Figure 3C depicts the value of linewidth Δ of the ST-FMR spectra as a function of *I*_dc_ in the range from −8 to 8 mA for the sample with *t*_i_ = 0.5 nm. The linewidths were varied in proportion to *I*_dc_. From the curve fitting of Fig. 3C with eq. S6, we obtained ξ_DL_ = 0.66 ± 0.07, whose magnitude is comparable to the value measured for a Pt(10)/Ni_95_Cu_5_(10) bilayer film (ξ_DL_ = 0.21 ± 0.01). Figure 3D shows ξ_DL_ as a function of *t*_i_. It is noted that the ξ_DL_ for the Pt/Ni_95_Cu_5_ bilayer is larger than the typical value of the spin Hall angle for Pt. Similar overestimation of ξ_DL_ has been commonly reported for W/CoFeB, Pt/CoFeB, and Pt/NiFe bilayers when the Gilbert damping modulation because of the dc current application is evaluated (*35*, *36*). Here, we mainly discuss the influence of the compositional gradient interface between Si and Al on the relative change of ξ_DL_. Compared to the sample without an Al/Si insertion layer (*t*_i_ = 0 nm), samples with *t*_i_ between 0.25 and 0.75 nm show a substantial enhancement in ξ_DL_. The sample with *t*_i_ = 0.5 nm achieves a peak ξ_DL_, approximately six times higher than that of the sample without the insertion layer. After this peak at *t*_i_ = 0.5 nm, ξ_DL_ decreases steadily. For *t*_i_ values exceeding 1.0 nm, we observed a modest increase in ξ_DL_, potentially because of the segregation of Si or Al at the interface, as shown in Fig. 2D of the HAADF-STEM image. This segregation suggests a possible qualitative change in interfacial structure, although the precise mechanism remains under investigation. We also evaluated the FL torque (see Supplementary Text S2). Although precision is limited, its trend is similar to that of the DL torque, with a notable peak at a 0.5-nm insertion layer. While further investigation into the origin of the FL torque is ongoing, our primary contribution lies in demonstrating that the independently determined DL torque efficiency in the Si/Al gradient material is comparable to or surpasses that of the SHE in Pt, irrespective of the FL torque efficiency. This result represents a notable advancement in spin torque materials development and indicates a promising route for materials with enhanced spin torque properties.

### Origin of spin torque accompanied by Si/Al gradient materials

Below, we discuss the origin of spin torque in the Si/Al/Ni_95_Cu_5_ trilayer film. Similarly to a torque generated via SHE and/or REE with an electric current along the *x* axis, the variations of *V*_s_ and *V*_a_ with respect to the direction of the external magnetic field can be explained by assuming that the electron spin is polarized along the *y* axis (see Supplementary Text S3) (*37*–*39*). In the Si/Al/Ni_95_Cu_5_ trilayer film, most of the electric current flows not within the Si or Ni_95_Cu_5_ layer but in the Al layer because the electric conductivity of Al is much larger. However, the spin torque produced by the electric current flow in the Al layer via SHE can be ignored because the spin Hall angle for bulk Al is only 0.02 (*8*, *40*), which is 33 times smaller than the value of ξ_DL_ in our sample with *t*_i_ = 0.5 nm.

One plausible explanation for the increase in ξ_DL_ is spin current generation via SVC (*23*, *24*, *41*). Because of conductivity differences between Si and Al, a nonuniform current forms in the nanometer-scale gradient layer. Supplementary Text S4 shows that the increase in ξ_DL_ correlates with SVC theory–based models. However, our simple model diverges as *t*_i_ → 0 nm and thus does not directly apply at atomic scales. In addition, surface and interface scattering within the Al layer may produce nonuniform current, resulting in a gradient in current density and opposite vorticity signs between the top and bottom surfaces. This mechanism aligns with SVC and is consistent with prior studies on SVC in fluid systems (*23*). Atomic-scale interfacial disturbance caused by sputter deposition with a small *t*_i_ increases electron scattering, altering the vorticity gradient distribution. The interplay between increased interfacial scattering and a reduced compositional gradient with increasing *t*_i_ may produce a peak in spin torque efficiency at an optimum *t*_i_. The dependence of ξ_DL_ on *t*_i_ could, in part, reflect this mechanism. To demonstrate SC generation via SVC, it is crucial to clarify how current distribution varies with *t*_i_.

Furthermore, the nonmonotonic variation in ξ_DL_ for small *t*_i_ values may stem from the irregular interfacial mixing on Al and Si at atomic scales. Okano *et al.* (*29*) observed nonmonotonic spin current generation efficiency in oxidized Cu, attributed to variations in oxidation stages. Similar interfacial mixing behavior may apply here. Consequently, while SVC provides reasonable explanation mechanisms, further investigation is required to understand spin current generation mechanisms fully, including nonmonotonic variations.

We must also consider the impact of inversion symmetry breaking (ISB) at the Si/Al interface, as the REE and orbital-REE (*42*–*47*), both induced by ISB, can enhance spin torque. Increasing the insertion layer thickness expands the volume of the Si/Al compositional gradient interface but also blurs the interface, reducing the magnitude of ISB. To discuss the *t*_i_ dependence of spin torque efficiency, it is essential to account for these interactions. However, determining the *t*_i_ dependence of ISB strength is challenging, as investigating the electronic structure of compositional gradient interfaces via first-principles calculations is not straightforward.

### Highly nonreciprocal SC generation in Si/Al gradient materials

In the Si/Al gradient materials, we observe pronounced nonreciprocal conversion between electric current and SC, which has been reported in other gradient materials consisting of CuO*_x_* (*29*) and Ti/W (*48*). This nonreciprocity may represent a general characteristic of spin current generation in gradient structures and could offer valuable insights into the underlying mechanisms of spin current generation in these systems.

**Fig. 4.**
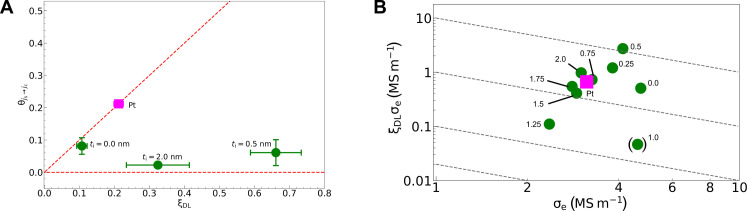
Nonreciprocal conversion between charge and spin current and FOM for Si/Al gradient material. (**A**) Conversion efficiency from SC to charge current $\theta_{j_{s}\to j_{c}}$ as a function of ξ_DL_. The circles show data for samples with various *t*_i_’s, whereas the square represents values for a Pt(10)/Ni_95_Cu_5_(10) bilayer film. The vertical bars indicate the standard deviation calculated from the least-squares deviation of the fitting parameters used to calculate $\theta_{j_{s}\to j_{c}}$. (**B**) Double-logarithmic plot of electric conductivity σ_e_ and $\xi_{\text{DL}}\sigma_{e}$ for samples with *t*_i_ ranging from 0 to 2.0 nm. The numbers next to the plots represent the *t*_i_ values in unit of nm. The data for a Pt(10)/Ni_95_Cu_5_(10) bilayer film are also plotted for comparison (square). Dashed lines are contours of $\xi_{\text{DL}}\sigma_{e}^{2}$.

To evaluate the conversion efficiency from SC to electric current, as shown in Supplementary Text S5, we measured the inverse SHE resulting from the SC (*49*, *50*), which was produced by applying an alternating magnetic field (*51*–*54*). Figure 4A shows the conversion efficiency $\theta_{j_{s}\to j_{c}}$ of the SC to an electric current as a function of ξ_DL_, which is proportional to the conversion efficiency $\theta_{j_{c}\to j_{s}}$ from the charge current to the SC. In general, an SC source with a two-dimensional geometry, such as an interfacial SOI, suppresses $\theta_{j_{s}\to j_{c}}$ rather than $\theta_{j_{c}\to j_{s}}$ by a factor of 5 or more (*55*, *56*). If the SC source does produce such a geometrical effect in the Si/Al gradient material, the nonreciprocity is expected to be less than 5 because of its fuzzy interface. However, as shown in Fig. 4A, the sample with *t*_i_ = 0.5 nm exhibits a $\xi_{\text{DL}}∕\theta_{j_{s}\to j_{c}}$ value larger than 5. On the other hand, $\xi_{\text{DL}}∕\theta_{j_{s}\to j_{c}}$ for *t*_i_ = 0 nm is unity, at which the highest two-dimensional geometry is expected.

One possible explanation for this pronounced nonreciprocity is SC generation from macroscopic vorticity in the electric current at the compositional gradient, mediated by SVC. The Berry curvature acts as an effective magnetic field, producing a spin-dependent force that is always perpendicular to the electron’s momentum because of isotropic scattering from SOI. This effect allows for reciprocal conversion between charge and spin currents regardless of the presence of an external electric field. In contrast to the Berry curvature, where the emergent magnetic field originating from the electron structure persists even when the electric field is zero, the emergent magnetic field in the SVC mechanism, which depends on current vorticity, vanishes when the electric field and current perpendicular to the conductance gradient are absent. The SVC mechanism relies on a vorticity gradient to generate spin-dependent forces directed along this gradient. When an in-plane charge current is applied, a vorticity gradient forms in the *z*-direction as illustrated in Fig. 1, creating spin-dependent forces along the *z* axis and thus enabling charge-to-spin conversion, as observed in the ST-FMR experiments. However, when a spin current is injected along the *z* axis, no in-plane charge current (or associated vorticity gradient) exists to produce spin-dependent forces, thereby preventing spin-to-charge conversion, as seen in the spin pumping experiments. The nonreciprocity observed in our study is, therefore, profoundly influenced by the presence of the vorticity gradient, which contributes to spin-dependent scattering, as well as the relative orientation between this gradient and the incident current.

### FOM for spin torque switching capability of Si/Al gradient materials

To reduce the supply voltage required for spin torque switching, it is crucial to enhance the product of ξ_DL_ and the electrical conductivity σ_e_ of the NM layer. A higher σ_e_ also helps mitigate resistive-capacitive delays that can obstruct high-speed operation of integrated circuits. Figure 4B shows a double-logarithmic plot of $\xi_{\text{DL}}\sigma_{e}$ versus σ_e_ for our Si/Al gradient samples. On the basis of these considerations, we define a FOM for SC-generating materials as

$$\begin{array}{c} \text{FOM}=\left(\xi_{\text{DL}}\sigma_{e}\right)\times \sigma_{e}\\ =\left[\text{Spin torque efficiency}\right]\times \left[\text{Circuit performance}\right] \end{array}$$ (3)

We also include data for a Pt/Ni_95_Cu_5_ bilayer in Fig. 4B (see the closed square). The dependence of σ_e_ on the insertion-layer thickness *t*_i_ is detailed in Supplementary Text S7. The dashed contours in Fig. 4B correspond to FOM = ξ_DL_$\sigma_{e}^{2}$. By reducing *t*_i_ from 2.0 to 0.5 nm in Si/Al gradient films, we can boost σ_e_ with increasing $\xi_{\text{DL}}\sigma_{e}$. This is unusual because strong-SOI materials (e.g., Pt, W, and Ta) typically show low σ_e_. For *t*_i_ = 0.5 nm, ξ_DL_$\sigma_{e}^{2}$ reaches 11.2 × 10^12^ (S/m)^2^, five times higher than the value for Pt.

Furthermore, to assess power consumption in spin-orbit torque magnetic random-access memory, we use the formula from (*57*, *58*)

$$P_{\text{write}}\propto {\left(\frac{1+s}{\xi_{\text{DL}}}\right)}^{2}\sigma_{e}^{-1}$$ (4)

where *s* is the fraction of current shunted through the FM layer, derived from the resistivity ratio. Our measurements indicate that the write power for the sample with *t*_i_ = 0.5 nm is less than 110 that of Pt, demonstrating the exceptional energy efficiency of our Si/Al gradient material. Consequently, they can serve as “green materials” for low-power applications.

While our FOM emphasizes performance metrics, we also consider the relative abundance *P*_n_ of the constituent elements as an additional indicator of sustainability. According to the data in Fig. 1A, *P*_Si/Al_ = *P*_Si_ × *P*_Al_ = 2.24 × 10^−2^, roughly seven orders of magnitude larger than that of Pt (5 × 10^−9^). This highlights the potential of the Si/Al gradient for resource-friendly spintronic development. Crucially, the underlying mechanism driving spin current generation in these films does not rely on heavy-element spin-orbit coupling, broadening the design space for advanced, sustainable spin devices.

## DISCUSSION

In this study, we explored an efficient charge-to-spin conversion mechanism in Si/Al compositional gradient materials, focusing on their potential for spintronic devices. By controlling the gradient width, we achieved a pronounced increase in spin torque efficiency (ξ_DL_), highlighting how gradient optimization can facilitate spin current generation.

A notable aspect of Si/Al gradient materials is their use of silicon and aluminum—two widely available, environmentally friendly elements. While this benefit might be less critical for certain device architectures that still rely on rare metals (e.g., in magnetic tunnel junctions), the introduction of Si/Al as a more “sustainable” material option broadens the landscape for spin current–based systems.

Crucially, these materials generate spin currents without the heavy reliance on strong SOI, thus providing greater flexibility in device design. Our FOM analysis reveals that Si/Al gradient films can match—or even surpass—the performance of Pt-based structures, not only in spin torque efficiency but also in energy efficiency. Measurements show a substantial reduction in write power relative to Pt, underscoring their potential for low-power spintronics.

Looking forward, these findings suggest that compositional gradient strategies could be extended to other material systems. By leveraging abundant elements like Si and Al, we present a promising pathway to high spin torque efficiency while maintaining an eye toward resource sustainability. This balance between performance and environmental consideration marks a promising direction for next-generation spintronic devices.

## MATERIALS AND METHODS

### Sample preparation

The films were fabricated on thermally oxidized silicon substrates by magnetron sputtering at room temperature. The chamber base pressure before deposition was less than 5.0 × 10^−4^ Pa. The deposition pressure was 0.22 Pa with an argon (Ar) flow rate of 4.0 standard cubic centimeters per minute. The Al layer creation used radio frequency (RF) deposition at 13.56 MHz with a power density of 1.4 W/m^2^ and a deposition rate of 0.043 nm/s from a 99.9% pure Al target. The Si layer was deposited by RF sputtering with a power density of 3.5 W/m^2^ and a deposition rate of 0.062 nm/s from a Si target. The NiCu layer was deposited by dc sputtering with a power density of 1.4 W/m^2^ and a deposition rate of 0.2 nm/s from a 99.9% pure Ni_95_Cu_5_ alloy target. The SiO_2_ capping layer was deposited by RF sputtering with a power density of 3.5 W/m^2^ and a deposition rate of 0.044 nm/s from a 99.99% pure SiO_2_ target. The thin films were patterned into 10-μm-wide and 100-μm-long strips by photolithography and liftoff processes.

An electrically shorted coplanar waveguide made from 70-nm-thick Au was connected to both ends of the strip to conduct the ST-FMR measurements. All measurements were conducted at room temperature.

### Electrical measurements

For a dc Gilbert damping modulation of the ST-FMR spectrum, we applied 20-dBm continuous sinusoidal signals with a frequency of 20 GHz in the longitudinal direction (*x* axis) of the film by a signal generator. An in-plane external magnetic field was applied with an amplitude ranging from 0 to 2.0 T at a fixed angle of π/4 or 5π/4 with respect to the *x* axis. For the linewidth modulation, we varied the dc in the range between −8 and 8 mA. We then measured the rectified dc voltage *V*_dc_ by a nanovoltmeter from a dc port of bias tee. The resulting spectra were fit by the expression *V*_dc_ = *V*_s_*f*_s_(*B*) + *V*_a_*f*_a_(*B*), where *f*_s_(*B*) and *f*_a_(*B*) are the symmetric and antisymmetric Lorentzian functions, respectively. The inverse SHE measurement used a general spin-pumping experiment setup. We applied a continuous sinusoidal signal with an amplitude of 20 dBm and a frequency of 5 GHz into the coplanar waveguide fabricated on the sample, which was patterned into a Hall-bar shape. The sample and coplanar waveguide were insulated by the insertion of a 120-nm-thick SiO_2_ film. The external magnetic fields with amplitudes ranging from 0 to 2.0 T were applied in the *x-y* plane at an angle ranging between 0 and 2π from the *x* axis. The dc inverse spin Hall voltage was measured using a nanovoltmeter.

### Electron microscopy characterization

We prepared cross-sectional thin specimens for HAADF-STEM characterization using a focused ion beam with a Ga^+^ ion source on a FEI Helios G4 UX instrument. Before the milling process, a ~5-nm-thick Au layer was deposited on the film surface to protect and enhance the conductivity of samples during milling. The lift-out lamellae were thinned to ~100 nm at 30 kV with the current decreasing from 0.75 nA to 90 pA, followed by final polishing at 2 kV and 17 pA. HAADF-STEM and nanobeam electron diffraction images were obtained using a Cs-corrected FEI Titan G2 80-200 equipped with a Super-X EDS. The HAADF-STEM images were collected using a convergence semiangle of 18 mrad and an inner-collection semiangle of 55 mrad. EDS mapping was performed using a Bruker Esprit analysis system with automatic drift correction during the collection process, which augments the reliability of the compositional analysis. Integrated line profiles were conducted across the heterostructure in EDS maps to enhance the signal-to-noise ratio. The Cliff-Lorimer analysis method was applied to quantify the EDS line-scan results.

## Supplementary Materials

### This PDF file includes:

Supplementary Text S1 to S7 Table S1 Figs. S1 to S8 References
