# Supplementary material for: Nanometer-thick Si/Al gradient materials for spin torque generation
Source: Sci Adv. 2025 May 9;11(19):eadr9481. doi: 10.1126/sciadv.adr9481 (PMC12063650; doi:10.1126/sciadv.adr9481)
Supplement: Supplementary file 1 — Supplementary Text S1 to S7 Table S1 Figs. S1 to S8 References [file sciadv.adr9481_sm.pdf]

Science Advances
AAAS

Supplementary Materials for

Nanometer-thick Si/Al gradient materials for spin torque generation

Taisuke Horaguchi *et al.*

Corresponding author: Yukio Nozaki, nozaki@phys.keio.ac.jp

Sci. Adv. **11**, eadr9481 (2025)
DOI: 10.1126/sciadv.adr9481

This PDF file includes:

Supplementary Text S1 to S7
Table S1
Figs. S1 to S8
References

Supplementary Text

S1 Theory to evaluate $\xi_{\rm DL}$ using direct current Gilbert damping modulation of ST-FMR spectrum.

Figure S1A illustrates the principle of a direct current(DC) Gilbert damping modulation of ST-FMR spectrum along with the chosen coordinate system. An alternating current $j_{\rm rf}$ is applied to the NM/FM strip along the x-axis followed by a DC voltage, $V_{\rm DC}$, measurement along the x-axis as a function of the external DC field $\boldsymbol{B}$. The component of $j_{\rm rf}$ that flows within the NM produces an alternating Oersted field $h_{\rm Oe}$ along the y-axis, as well as a spin current j_s via SHE. An electron spin in j_s is polarized along the y-axis. Both $h_{\rm Oe}$ and j_s produce an alternating torque on the magnetization of the FM, which temporarily alters the electrical resistance of the FM through an anisotropic magnetoresistance (AMR) effect. Simultaneously, the $h_{\rm Oe}$ produces an alternating current in the FM via electromagnetic induction. A $V_{\rm DC}$ is subsequently produced in the FM by a rectification between the induced alternating current and the temporally varying electrical resistance. The spectrum of the $V_{\rm DC}$ with respect to $\boldsymbol{B}$ consists of symmetric and antisymmetric components that are described by Lorentzian and anti-Lorentzian functions, as follows (*31*):

$$V_{\rm DC} = V_{\rm s} f_{\rm s}(B) + V_{\rm a} f_{\rm a}(B) \tag{S1}$$

$$f_{\rm s}(B) = \frac{\Delta^2}{(B - B_{\rm r})^2 + \Delta^2}, \tag{S2}$$

$$f_{\rm a}(B) = \frac{\Delta(B - B_{\rm r})}{(B - B_{\rm r})^2 + \Delta^2}, \tag{S3}$$

where $B_{\rm r}$ and Δ are the ferromagnetic resonance field and the field-domain linewidth, respectively. $V_{\rm s}$ is the amplitude of the symmetric component although $V_{\rm a}$ is that of the antisymmetric one. Both $B_{\rm r}$ and Δ depend on the frequency f of $j_{\rm rf}$ as follows:

$$f = \frac{\gamma}{2\pi}\sqrt{B_{\rm r}(B_{\rm r} + \mu_0 M_{\rm s})}, \tag{S4}$$

$$\Delta = \frac{2\pi\mu_0\alpha_{\rm eff} f}{\gamma}, \tag{S5}$$

where $\alpha_{\rm eff}$ is the effective damping. Figure S2 shows the frequency dependence of $B_{\rm r}$ measured for the sample with $t_{\rm i}$ = 0.5 nm. From the best fit to Eq. (S4), the value of $M_{\rm s}$ for $Ni_{95}Cu_5$ is determined as 0.60 T, whose magnitude is similar to that expected from the Slater-Pauling curve. When a direct current $I_{\rm DC}$ is simultaneously applied with $j_{\rm rf}$, a damping-like (DL) torque due to a spin current produced by the $I_{\rm DC}$ modulates the $\alpha_{\rm eff}$ (*31, 32*).

The magnitude of the corresponding linewidth modulation, $\delta\Delta$, is given by,

$$\delta\Delta = \frac{2\pi f}{\gamma}\frac{\sin\varphi}{(B_{\rm r} + \mu_0 M_{\rm s}\,/\,2)M_{\rm s} d_{\rm FM}}\frac{\hbar}{2e}\xi_{\rm DL} j_{\rm DC}, \tag{S6}$$

where φ is the angle between $I_{\rm DC}$ (along the x axis) and magnetization $\boldsymbol{m}$. And the current density $j_{\rm DC}$ at the NM layer can be obtained from the relationship of $j_{\rm DC} = I_{\rm DC}\,/\,w d_{\rm NM}$, where w and $d_{\rm NM}$ are the strip line width and the thickness of NM layer, respectively. According to Eq.(S6), we can evaluate the value of $\xi_{\rm DL}$ from the slope of $\delta\Delta$ as a function of $j_{\rm DC}$. The direction of the spin transfer torque should be switched by reversing the spin polarization of the spin current or the magnetization. Indeed, as shown in Fig. 3C of the main text, the sign of the slope is switched when the direction of magnetization is reversed. In this article, the value of $\xi_{\rm DL}$ is evaluated by averaging the absolute values of the slope measured for positive and negative external magnetic fields.

S2 Evaluation of field-like torque efficiency

The amplitudes of V_s and V_a in Eq. (S1) are given by (*31, 34*),

$$V_s = \frac{\hbar}{2e}\frac{\xi_{DL} j_{rf}}{\mu_0 M_s d_{FM}}, \tag{S7}$$

$$V_a = \left(\frac{\hbar}{2e}\frac{\xi_{FL} j_{rf}}{\mu_0 M_s d_{FM}} + \frac{j_{rf} d_{NM}}{2}\right)\sqrt{1+\frac{\mu_0 M_s}{B_r}}, \tag{S8}$$

respectively. The first and second terms in Eq. (S8) are associated with the field-like (FL) torque and Oersted field torque, respectively. As shown in Eqs. (S7) and (S8), the rectified voltages associated with the DL and FL torques can be improved by decreasing M_s in comparison to that owing to the Oersted field torque. In this article, to improve the signal-to-noise ratio of the ST-FMR spectrum, Ni-Cu alloy, whose M_s can be reduced in comparison to the permalloy, has been used for observing the ST-FMR spectrum. It is also noted that the Gilbert damping constant of the Ni-Cu alloy is increased with increasing the compound of Cu, which makes it hard to capture the direct current modulation of Gilbert damping in the ST-FMR spectrum. We have chosen $Ni_{95}Cu_5$ as a detector of the spin torque to satisfy the conflicting demands. In the ST-FMR measurement, the magnitudes of both ξ_{DL} and ξ_{FL} are generally evaluated from the d_{FM} dependence of V_s / V_a. In the evaluation, it is convenient to use a parameter defined as follows (*31, 34*):

$$\xi_{FMR} = \frac{V_s}{V_a}\frac{e\mu_0 M_s d_{FM} d_{NM}}{\hbar}\left(1+\frac{\mu_0 M_s}{B_r}\right)^{\frac{1}{2}} \tag{S9}$$

$$= \xi_{DL}\left(1+\frac{\hbar}{\mu_0 M_s d_{FM} d_{NM}}\xi_{FL}\right)^{-1}. \tag{S10}$$

Figures S3A-E show ξ_{FMR}^{-1} as a function of $(\mu_0 M_s d_{FM})^{-1}$ measured for samples with $t_i = 0, 0.5, 1.0, 1.5$ and 2.0 nm, respectively. The dashed lines in Fig. S3 indicate the best-fit results for Eq. (S9), where the value of ξ_{DL} is fixed at that evaluated for each sample from the direct current modulation of the Gilbert damping constant as discussed in S1. Figure S4 shows the ξ_{FL}, which is evaluated from the curve fitting with Eq. (S10), as a function of t_i. Similar to the variation of ξ_{DL} with t_i, the value of ξ_{FL} is increased with decreasing t_i and is maximized at t_i = 0.5 nm. This result also supports the assumption that the nanometer-thick gradient from Si to Al is the most important factor for SC generation in the sample, although the reason why the magnitude of ξ_{FL} is several times larger than that of ξ_{DL} is still an open question. The FL torque conventionally arises from (i) spin accumulation at magnetic/non-magnetic interfaces due to the REE (*14, 59*), (ii) self-torque within the FM layer (*60, 61*), and (iii) the rotation of spins due to imperfect angular momentum transfer at the FM/NM interface (*59, 62*). Notably, the FL torque demonstrates a similar dependence on t_i as the DL torque, as shown in Fig. S4. In our experiments, the FM/NM interface of all Si/Al-based devices remains consistent for Ni-Cu/Al. Consequently, attributing the t_i dependency of the FL torque solely to REE is not feasible. Conversely, the magnitude of self-torque within the FM layer, proportional to the current flowing through it, varies with t_i due to the dependence of the electric conductivity on t_i. However, as illustrated in Fig. S8, the electrical conductivity of Si/Al gradient materials decreases with increasing t_i, thus failing to explain the observed maximum FL torque at t_i = 0.5 nm. We believe that the t_i dependence of the FL torque originates from t_i variation in the nonadiabatic spin transfer torque. The non-adiabatic component of spin transfer torque depends on the magnitude of the injected spin current at the interface because the spin scattering effect at the Ni-Cu/Al interface is independent of t_i. Consequently, the similar t_i dependence of DL torque and FL torque is not contradictory. The observation that FL

torque surpasses DL torque by several times suggests a notable effective field at the Al/Ni-Cu interface. We plan to investigate the origin of this phenomenon in future studies. Unfortunately, the cause of the increase in FL torque generation efficiency beyond 1 remains unidentified. Nonetheless, the primary contribution of this study lies in demonstrating that the independently determined DL torque efficiency equals or exceeds the bulk spin Hall effect of Pt, irrespective of FL torque efficiency. This constitutes a notable advancement, paving the way for novel material development to induce substantial spin torques.

S3 Angular dependence of ST-FMR signals

Here, we discuss the angular dependence of symmetric- and antisymmetric-Lorentzian components in ST-FMR measurements. When both the polarization $\boldsymbol{\sigma_s}$ of a spin current and the RF field h_{Oe} generated by $\boldsymbol{j}_{\mathrm{rf}}$ are parallel to the y-axis, i.e. transverse to the NM/FM strip, the amplitudes of both the symmetric- and antisymmetric-Lorentzian components, V_{s} and V_{a} , are known to be proportional to $\sin(2\varphi)\cos\varphi$ (*37–39*), where the $\sin(2\varphi)$ component is attributed to a change in the AMR effect, while the $\cos\varphi$ component describes the angular dependence of the torque amplitude exerted on magnetization. If $\boldsymbol{\sigma_s}$ and $\boldsymbol{h}_{\mathrm{Oe}}$ contain x- and z-components, the functions for the angular dependence of V_{s} and V_{a} are mixed with additional components of $\sin(2\varphi)\sin\varphi$ and $\sin(2\varphi)$, respectively. Consequently, the angular dependences of V_{s} and V_{a} are given by

$$V_{\mathrm{s(a)}} = V_{\mathrm{s(a)}}^{y}\sin(2\varphi)\cos\varphi + V_{\mathrm{s(a)}}^{z}\sin(2\varphi) + V_{\mathrm{s(a)}}^{x}\sin(2\varphi)\sin\varphi\,, \qquad \text{(S11)}$$

where $V_{\mathrm{s(a)}}^{y}$, $V_{\mathrm{s(a)}}^{z}$, and $V_{\mathrm{s(a)}}^{x}$ are the amplitudes of three different angular dependences. Table S1 summarizes the correlation between the origin of the torque on magnetization and the angular dependence of $V_{\mathrm{s(a)}}$. Previous studies predicted that angular dependences other than $\sin(2\varphi)\cos\varphi$ in Eq.(S11) appeared in the presence of the Rashba-type spin-orbit field, which leads to anisotropic spin relaxation. Moreover, a non-uniform distribution of j_{rf} also leads to full angular dependence, as shown in Eq. (S11). Figure S5 shows the angular dependence of V_{s} and V_{a} for the sample with $t_{\mathrm{i}} = 0.5$ nm, which exhibited the largest spin-torque efficiency. The thick solid curves in Fig. S5 indicate the best-fit result with Eq.(S11). From the curve fitting, we find that the $\sin(2\varphi)\cos\varphi$ component is dominant in both V_{s} and V_{a} . Namely, $\boldsymbol{\sigma_s}$ and $\boldsymbol{h}_{\mathrm{Oe}}$ are parallel to the y-axis. In other words, the polarization of the spin current generated in the Si/Al gradient material is similar to that of the general spin Hall effect caused by a bulk SOI.

S4 Rough estimate of spin-torque efficiency enhancement according to SVC theory

To examine the role of the compositional Si/Al gradient as a potential origin of augmented ξ_{DL}, the amplitude of SC via SVC was numerically evaluated according to the theory for SVC. According to the SVC theory (*29*), the magnitude of the SC source produced by an electric current vorticity is proportional to $(l/L)^2$, where l is the volume-averaged mean free path of a free electron in the Si/Al gradient material. In general, the mean free path of free electrons is proportional to the electric conductivity. Because Si and Al are an atomic combination in non-solid solutions, the electrical resistivity of the two-phase Si and Al mixture varies monotonously with respect to the mixing ratio. Local values of electric conductivity and the electron mean free path in the compositional gradient from Si to Al, therefore, vary in the same manner as the cross-sectional composition profile, as shown in Fig. 2 in the main text. The volume-averaged electron mean free path of the Si/Al gradient material was reduced to half of the value for Al and is independent of the transition width L. As a consequence, the magnitude of the SC source produced

by the electric current vorticity is inversely proportional to L^2. Figure 3D in the main text shows that ξ_{DL} increased from 0.22 to 0.66 when t_{i} was decreased from 0.75 to 0.5 nm. The increasing rate of ξ_{DL} of 3.0 is on the same order of magnitude as the value of (0.75 nm/0.5 nm)^2 ~ 2.3 expected from SVC theory. To precisely evaluate the spin-torque produced by the Si/Al gradient material, we need to refine the numerical model so that in addition to SVC, other effects associated with inversion symmetry breaking and interfacial SOI can be accurately determined for the compositional gradient.

S5 Evaluation of non-reciprocity in inter-conversion of charge and spin

Figures S6A,B show a schematic principle of the spin pumping effect and experimental setup for measuring the inverse spin Hall voltage V^{ISHE}. In this experiment, we fabricated the Hall bar shaped Si/Al/Ni-Cu strip followed by an electrically shorted coplanar waveguide (CPW) made of Au. The Oersted field generated by the current flowing through the CPW excites the FMR in Ni-Cu layer. Note that, since the strip and CPW are separated by insulating $\mathrm{SiO_2}$ layer, a spin current due to the spin Hall effect in Au does not impact to the ferromagnetic Ni-Cu in the strip. The excitation of FMR in the Ni-Cu leads to a spin accumulation at the interface between Al and Ni-Cu so that a spin current is diffused toward the Si/Al gradient material. If the Si/Al gradient material converts the spin current to a charge current via inverse SHE, a finite V^{ISHE} is measured. For instance, a direct spin current polarized along the x-axis is converted to a charge current which produces V^{ISHE}E along the y-axis. We measured the V^{ISHE} by yellow electrode when the rf current was applied into the green CPW in Fig. S6B. Figures S7A-C show the inverse spin Hall voltage spectra V^{ISHE} measured for Si / Al / $\mathrm{Ni_{95}Cu_5}$ trilayer films with $t_{\mathrm{i}} = 2.0$, 0.5 and 0 nm, respectively, at various angles of the external magnetic field. The microwave frequency used for the spin pumping was fixed at 5 GHz. As shown in Fig. S7D, a similar measurement was conducted for a $\mathrm{Pt(10)}$ / $\mathrm{Ni_{95}Cu_5(10)}$ bilayer film where a large inverse SHE is expected due to the strong SOI in the Pt. Figures S7E-H show the angular dependence of the amplitude of the symmetric Lorentzian component of V^{ISHE} for each t_{i}. The spin polarization of the pumped SC is parallel to the precession axis, which is consistent with the direction of the external DC magnetic field. An inverse SHE in the NM leads to V^{ISHE} produced by the SC. The magnitude of V^{ISHE} is proportional to $\cos 3\varphi$ (*37*), where φ is the angle of the external DC field from the x-axis. An applied AC magnetic field also produces an AC electric current in the metallic FM via electromagnetic induction. Moreover, the magnetization precession leads to a temporal change in the electrical resistance of the FM via the AMR effect. Consequently, a rectified DC photovoltage appears in the FM. Harder et al. reported that AC magnetic fields applied along the x-, y-, and z-axes produce DC photovoltages that are proportional to $\sin 2\varphi \sin\varphi$, $\sin 2\varphi \cos\varphi$ and $\sin 2\varphi$, respectively (*37*). Here, the AC magnetic fields along x- and y-axes are drevies from the misalignment of the CPW. Since these photovoltages are produced in the FM, the angular dependence of the Lorentzian component of the inverse spin Hall voltage, $V_{\mathrm{s}}^{\mathrm{ISHE}}$, is given by

$$V_{\mathrm{s}}^{\mathrm{ISHE}} = A_0 \cos^3\varphi + A_1 \sin 2\varphi\ \sin\varphi + A_2 \sin 2\varphi \cos\varphi + A_3 \sin 2\varphi\ , \qquad \text{(S12)}$$

where A_0 is a coefficient that corresponds to the conversion efficiency from the SC to the charge current in the NM, whereas A_1, A_2 and A_3 are associated with the rectified voltages in the FM. Indeed, as shown by the solid curves in Fig. S7E-H, the dependence of $V_{\mathrm{s}}^{\mathrm{ISHE}}$ is well fitted by Eq. (S12), although a component that shows another angular dependence is slightly superimposed in the sample with $t_{\mathrm{i}} = 0.5$ nm. From the best-fit results with Eq. (S12), we obtained values of A_0 as 3.85, 0.71, and 5.86 μV for $t_{\mathrm{i}} = 2.0$, 0.5, and 0 nm, respectively, and 40.6 μV for the Pt. The conversion efficiency $\theta_{j_{\mathrm{s}}\to j_{\mathrm{c}}}$ from SC to charge current can be evaluated from these values using

the following equation (*52–54*):

$$A_0 = \frac{w\theta_{j_s \to j_c}\lambda_{NM}\tanh(d_{NM}/2\lambda_{NM})}{\sigma_{NM}d_{NM} + \sigma_{FM}d_{FM}}\frac{2e}{\hbar}j_s^{NM}, \tag{S13}$$

where w is the sample width. λ_{NM} and σ_{NM} (σ_{FM}) are the spin diffusion length and the electric conductivity of NM (FM), respectively. j_s^{NM} is the SC density at the NM/ $Ni_{95}Cu_5$ interface, whose magnitude is proportional to the real part of the spin-mixing conductance $g_r^{\uparrow\downarrow}$ at the interface. As shown in Supplementary note S7, this $g_r^{\uparrow\downarrow}$ can be evaluated from the increase in the Gilbert damping constant when the FM is attached to the NM. In the bilayer film with NM = Pt, the direct and inverse conversion efficiencies between the charge current and SC are expected to be identical, namely, $\theta_{j_s \to j_c} = \theta_{j_c \to j_s}$. In the direct current modulation experiment, ξ_{DL} has already been evaluated for the Pt/ $Ni_{95}Cu_5$ bilayer film. For simplicity, we neglect a reduction of V^{ISHE} due to an insufficient spin mixing conductance or a spin memory loss at the FM/NM interface. This assumption is reasonable from the fact that we have successfully measure the direct current Gilbert damping modulation of the ST-FMR spectrum, which is also affected by the spin mixing conductance and spin memory loss at the interface. Consequently, the magnitude of j_s^{NM} in the Pt/ $Ni_{95}Cu_5$ bilayer film can be evaluated from A_0 by substituting $\theta_{j_s \to j_c}$ in Eq. (S13) by ξ_{DL}. The magnitude of j_s^{NM} in the compositional gradient materials can be expected from the j_s^{NM} value with NM=Pt because j_s^{NM} is generally proportional to $g_r^{\uparrow\downarrow}$ as follows (*52*):

$$j_s^{NM} = g_r^{\uparrow\downarrow} f\hbar P\left(\frac{\gamma\mu_0 h_{Oe}}{2\alpha\omega}\right), \tag{S14}$$

$$P = \frac{2\omega\left[\gamma\mu_0 M_s + \sqrt{(\gamma\mu_0 M_s)^2 + 4\omega^2}\right]}{(\gamma\mu_0 M_s)^2 + 4\omega^2}. \tag{S15}$$

Here, ω is an angular frequency, which satisfying $\omega = 2\pi f$. Finally, as shown in Fig. 4A in the main text, the magnitude of $\theta_{j_s \to j_c}$ as a function of ξ_{DL} in the composition gradient materials can be evaluated from the j_s^{NM} value.

S6 Evaluation of spin-mixing conductance for an Al / $Ni_{95}Cu_5$ interface

In the main text, we demonstrate a strong non-reciprocity in the conversion efficiencies between the SC and charge current. The conversion efficiency from the SC to charge current $\theta_{j_s \to j_c}$ can be evaluated from the angular dependence of the inverse spin Hall voltage caused by spin pumping as shown in Supplementary note S6. To obtain the value of $\theta_{j_s \to j_c}$ from the inverse spin Hall voltage, we need to determine the real part of the spin-mixing conductance $g_r^{\uparrow\downarrow}$, which is associated with spin transport efficiency at the NM/FM interface. Here, we explain the method for evaluating $g_r^{\uparrow\downarrow}$ from the linewidth broadening of the ST-FMR spectrum. First, we measure the effective damping constants for both the Pt / $Ni_{95}Cu_5$ bilayer and the Si / Al / $Ni_{95}Cu_5$ trilayer films from the frequency dependence of the FMR linewidth. The effective damping is generally given by the following equation:

$$\alpha_{NM/Ni_{95}Cu_5}^{eff} = \frac{\gamma\Delta}{2\pi\mu_0 f}, \tag{S16}$$

where γ and Δ are the gyromagnetic ratio and the field-domain FMR linewidth, respectively. The $g_r^{\uparrow\downarrow}$ is evaluated from the $\alpha_{NM/Ni_{95}Cu_5}^{eff}$ by the following equation (*52–54*):

$$g_{r,NM/Ni_{95}Cu_5}^{eff} = \frac{\mu_0 M_s d_{FM}}{g\mu_B}\left(\alpha_{NM/Ni_{95}Cu_5}^{eff} - \alpha_{Ni_{95}Cu_5}^{eff}\right), \tag{S17}$$

where g and μ_B are the g-factor and Bohr magneton, respectively. $\alpha^{\mathrm{eff}}_{\mathrm{Ni_{95}Cu_5}}$ is the effective damping constant for the $Ni_{95}Cu_5$ monolayer film. Finally, we obtained a $g_r^{\uparrow\downarrow}$ value for the Pt / $Ni_{95}Cu_5$ bilayer film of $(6.0 \pm 1.7) \times 10^{18}$ $\mathrm{m^{-2}}$. In the case of Si / Al / $Ni_{95}Cu_5$ trilayer films, the values of $g_r^{\uparrow\downarrow}$ are estimated to be $(2.8 \pm 0.5) \times 10^{18}$ $\mathrm{m^{-2}}$, $(4.73 \pm 3) \times 10^{17}$ $\mathrm{m^{-2}}$, and $(2.9 \pm 0.7) \times 10^{18}$ $\mathrm{m^{-2}}$ for t_i = 2.0, 0.5, and 0 nm, respectively.

S7 Electric conductivity of Si/Al composition gradient materials vs. thickness of the interfacial Al/Si insertion

Figure S8 shows the electric conductivity σ_e for Si / Al / $Ni_{95}Cu_5$ trilayer films as a function of t_i . The values of σ_e in Fig. S8 are evaluated for $\mathrm{Si(10)/Al}(t_i/2)\mathrm{/Si}(t_i/2)\mathrm{/Al(10)/Ni_{95}Cu_5(10)/SiO_2(20)}$ by assuming a parallel circuit with the $Ni_{95}Cu_5$ (10 nm) channel, whose electric conductivity is 3.7 $\mathrm{MSm^{-1}}$. A thicker compositional gradient from Si to Al leads to a smaller σ_e. This result suggests that electron scattering at the Si/Al interface is enhanced by the compositional gradient and affects the electrical conductivity of the entire film.

Table S1 | Summary of relationships between the origin of the torque on magnetization and the angular dependence of V_s and V_a.

Angular-dep.	V_s	V_a
$\sin 2\varphi \cos\varphi$	$\mathrm{DL}(\sigma_y)$	$\mathrm{FL}(\sigma_y),\ h_{\mathrm{Oe},y}$
$\sin 2\varphi$	$\mathrm{FL}(\sigma_z),\ h_{\mathrm{Oe},z}$	$\mathrm{DL}(\sigma_z)$
$\sin 2\varphi \sin\varphi$	$\mathrm{DL}(\sigma_x)$	$\mathrm{FL}(\sigma_x),\ h_{\mathrm{Oe},x}$

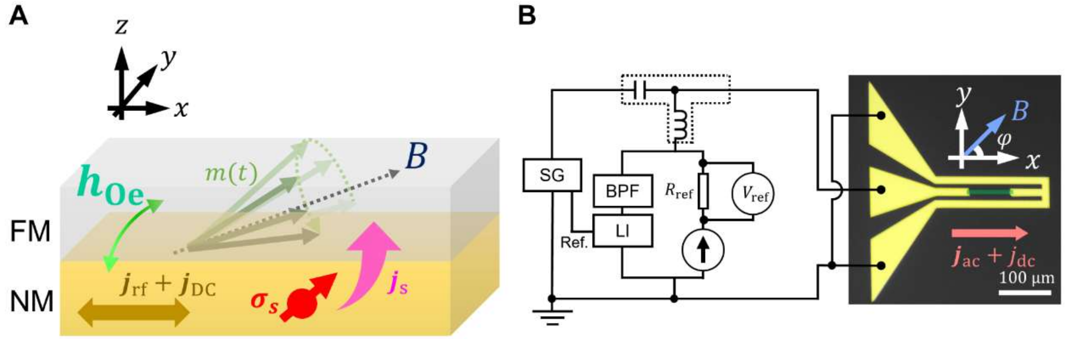

Fig. S1 | Spin-torque evaluation by a direct current damping modulation of ST-FMR experiment.

A, Schematic principle of a direct current damping modulation of ST-FMR, in which a ferromagnetic resonance in FM is excited by a spin-transfer torque from an electrical current-induced spin current in NM. **B**, Schematic configuration of the ST-FMR measurement, including the coordinate system used in this article.

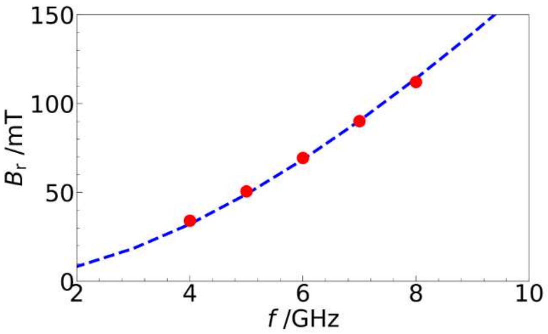

Fig. S2 | Resonant field as a function of microwave frequency.

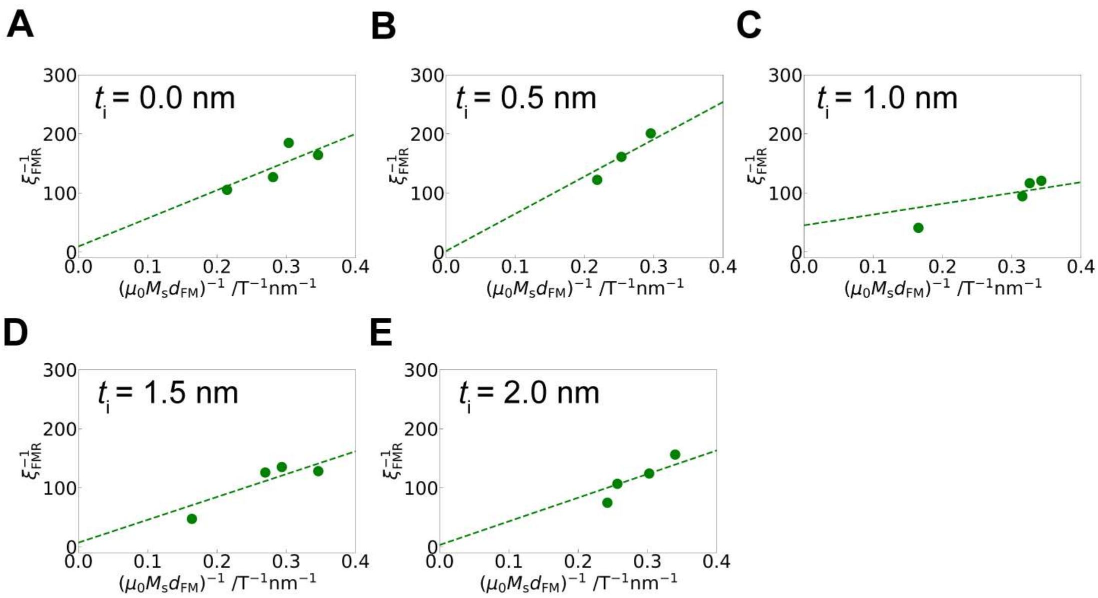

Fig. S3 |FM layer thickness dependence of ξ_{FMR}.
A-E, ξ_{FMR}^{-1} as a function of $(\mu_0 M_s d_{FM})^{-1}$ for the sample with **A** $t_i = 0.0$, **B** 0.5, **C** 1.0, **D** 1.5, **E** 2.0 nm, respectively.

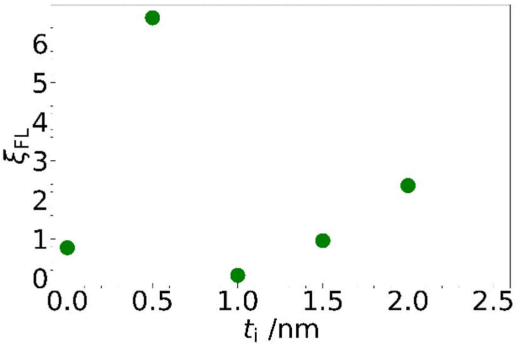

Fig. S4 | Insertion layer thickness dependence of ξ_{FL}.

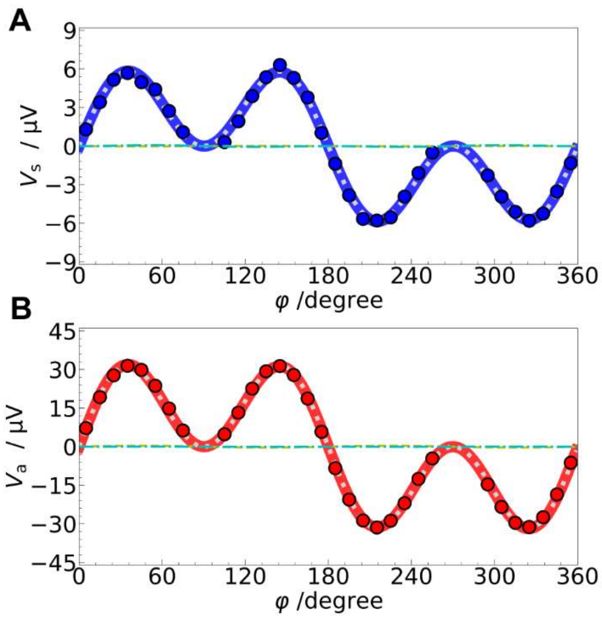

Fig. S5 | Angular dependence of ST-FMR spectrum amplitudes.

A,B Angular dependence of amplitudes in symmetric and antisymmetric Lorentzian components, **A** V_s and **B** V_a , measured for Si/Al composition gradient materials with t_i = 0.5 nm, which shows a largest spin-torque efficiency. φ is the angle of the applied external field from the x-axis.

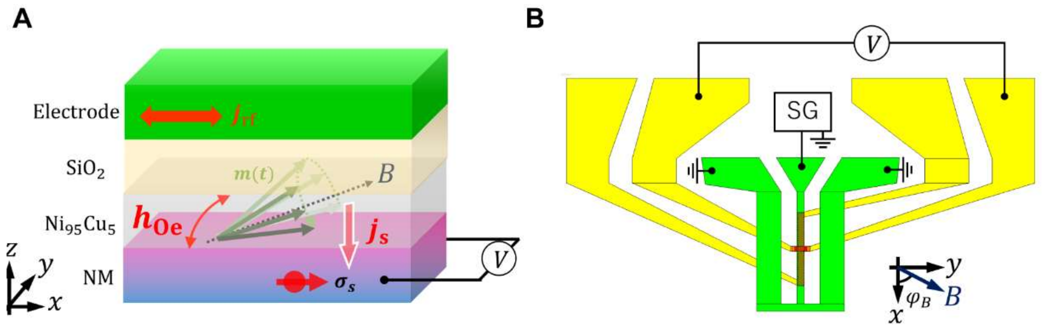

Fig. S6 | Spin to charge conversion efficiency evaluation by a spin-pumping experiment. **A**, Schematic principle of spin pumping. First, a ferromagnetic resonance in FM is excited by an Oersted field h_{Oe} generated from a current flowing through the top electrode. When the external bias field is applied along the y axis, a spin current with spin polarization parallel to y is injected into the adjacent NM layer due to the dissipation of angular momentum of FMR. If the injected spin current is converted into charge current by ISHE in the NM layer, a DC voltage is detected along the y-axis. **B**, Schematic configuration of the spin-pumping experiment, including the coordinate system used in this article. We applied microwaves of power 20 dBm to the green CPW, and we measured a DC voltage V^{ISHE} along the transverse direction of strip line by the yellow electrode. Here, the green CPW and NM / $Ni_{95}Cu_5$ strip line is insulated by a 20 nm of SiO_2 layer. We investigated the φ dependence of V^{ISHE}.

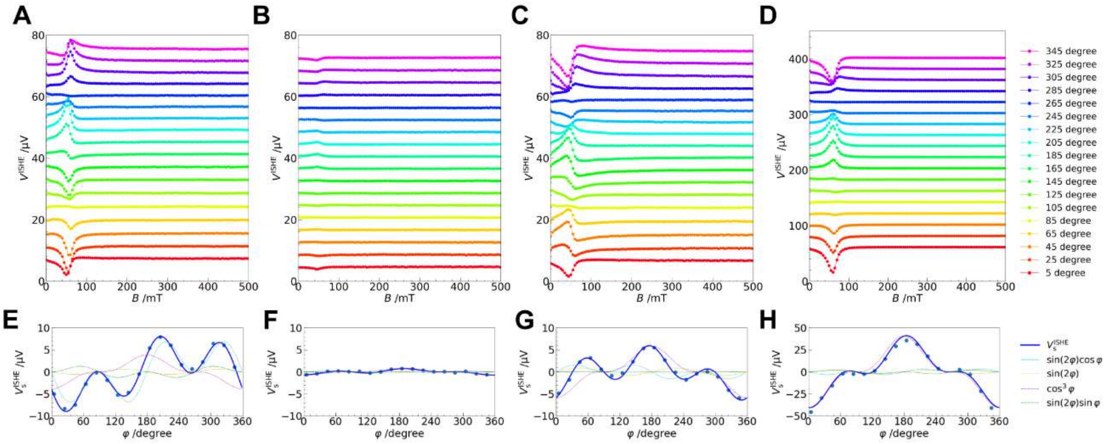

Fig. S7 | Characteristics of conversion from spin to charge currents.

A,B Inverse spin Hall voltage spectra measured at various angles of the applied external field for Si / Al / $Ni_{95}Cu_5$ trilayer films with **A** 2.0 nm and **B** 0.5 nm-thick Al/Si insertion at the Si/Al interface. The microwave frequency used for spin pumping was fixed at 5 GHz. **E,F** Angular dependence of the amplitude of the Lorentzian component of the inverse spin Hall voltage spectrum, V^{ISHE}, for each thickness. For comparison, similar data measured for Si / Al / $Ni_{95}Cu_5$ trilayer films without interfacial Al/Si insertion are shown in **C** and **G**. Moreover, the data measured for a Pt / $Ni_{95}Cu_5$ bilayer film are also shown in **D** and **H**.

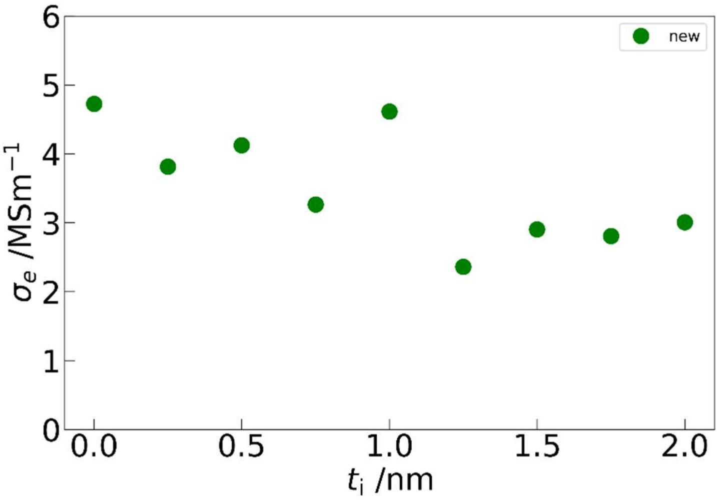

Fig. S8 | Electric conductivity of Si / Al / $Ni_{95}Cu_5$ trilayer films as a function of t_i.

REFERENCES AND NOTES

1. C. Chappert, A. Fert, F. N. Van Dau, The emergence of spin electronics in data storage. *Nat. Mater.* **6**, 813–823 (2007).

2. B. Tudu, A. Tiwari, Recent developments in perpendicular magnetic anisotropy thin films for data storage applications. *Vacuum* **146**, 329–341 (2017).

3. L. Liu, C.-F. Pai, D. C. Ralph, R. A. Buhrman, Magnetic oscillations driven by the spin Hall effect in 3-terminal magnetic tunnel junction devices. *Phys. Rev. Lett.* **109**, 186602 (2012).

4. V. E. Demicov, S. Urazhdin, H. Ulrichs, V. Tiberkevich, A. Slavin, D. Baither, G. Schmitz, S. O. Demokritov, Magnetic nano-oscillator driven by pure spin current. *Nat. Mater.* **11**, 1028–1031 (2012).

5. Z. Duan, A. Smith, L. Yang, B. Youngblood, J. Lindner, V. E. Demidov, S. O. Demokritov, I. N. Krivorotov, Nanowire spin torque oscillator driven by spin orbit torques. *Nat. Commun.* **5**, 5616 (2014).

6. J. E. Hirsch, Spin Hall effect. *Phys. Rev. Lett.* **83**, 1834–1837 (1999).

7. S. Zhang, Spin Hall effect in the presence of spin diffusion. *Phys. Rev. Lett.* **85**, 393–396 (2000).

8. S. O. Valenzuela, M. Tinkham, Direct electronic measurement of the spin Hall effect. *Nature* **442**, 176–179 (2006).

9. S. Maekawa, A flood of spin current. *Nat. Mater.* **8**, 777–778 (2009).

10. Y. K. Kato, R. C. Myers, A. C. Gossard, D. D. Awschalom, Observation of the spin Hall effect in semiconductors. *Science* **306**, 1910–1913 (2004).

11. V. M. Edelstein, Spin polarization of conduction electrons induced by electric current in two-dimensional asymmetric electron systems. *Solid State Commun.* **73**, 233–235 (1990).

12. S. D. Ganichev, E. L. Ivchenko, V. V. Bel'kov, S. A. Tarasenko, M. Sollinger, D. Weiss, W. Wegscheider, W. Prettl, Spin-galvanic effect. *Nature (London)* **417**, 153–156 (2002).

13. J. C. Rojas Sanchez, L. Vila, G. Desfonds, S. Gambarelli, J. P. Attane, J. M. De Teresa, C. Magén, A. Fert, Spin-to-charge conversion using Rashba coupling at the interface between non-magnetic materials. *Nat. Commun.* **4**, 2944 (2013).

14. A. Manchon, H. C. Koo, J. Nitta, S. M. Frolov, R. A. Duine, New perspectives for Rashba spin–orbit coupling. *Nat. Mater.* **14**, 871–882 (2015).

15. L. Liu, C.-F. Pai, Y. Li, H. W. Tseng, D. C. Ralph, R. A. Buhrman, Spin-torque switching with the giant spin Hall effect of tantalum. *Science* **336**, 555–558 (2012).

16. G. Allen, S. Manipaturuni, D. E. Nikonov, M. Doczy, I. A. Young, Experimental demonstration of the coexistence of spin Hall and Rashba effects in β-tantalum/ferromagnet bilayers. *Phys. Rev. B* **91**, 144412 (2015).

17. R. Yu, B. F. Miao, L. Sun, Q. Liu, J. Du, P. Omelchenko, B. Heinrich, W. Mingzhong, H. F. Ding, Determination of spin Hall angle and spin diffusion length in β-phase-dominated tantalum. *Phys. Rev. Mater.* **2**, 074406 (2018).

18. C.-F. Pai, L. Liu, Y. Li, H. W. Tseng, D. C. Ralph, R. A. Buhrman, Spin transfer torque devices utilizing the giant spin Hall effect of tungsten. *Appl. Phys. Lett.* **101**, 122404 (2012).

19. Q. Hao, G. Xiao, Giant spin Hall effect and switching induced by spin-transfer torque in a W/ $Co_{40}Fe_{40}B_{20}$/MgO structure with perpendicular magnetic anisotropy. *Phys. Rev. Appl.* **3**, 034009 (2015).

20. W. Gerlach, O. Stern, Der experimentelle nachweis der richtung-squantelung im magneticfield. *Z. Phys.* **9**, 349–352 (1922).

21. W. Gerlach, O. Stern, Das magnetische moment des silber atoms. *Z. Phys.* **9**, 353–355 (1922).

22. M. Matsuo, Y. Ohnuma, S. Maekawa, Theory of spin hydrodynamic generation. *Phys. Rev. B* **96**, 020401(R) (2017).

23. R. Takahashi, M. Matsuo, M. Ono, K. Harii, H. Chudo, S. Okayasu, J. Ieda, S. Maekawa, E. Saitoh, Spin hydrodynamic generation. *Nat. Phys.* **12**, 52–56 (2016).

24. D. Kobayashi, T. Yoshikawa, M. Matsuo, R. Iguchi, S. Maekawa, E. Saitoh, Y. Nozaki, Spin current generation using a surface acoustic wave generated via spin-rotation coupling. *Phys. Rev. Lett.* **119**, 077202 (2017).

25. Y. Kurimune, M. Matsuo, S. Maekawa, Y. Nozaki, Highly nonlinear frequency-dependent spin-wave resonance excited via spin-vorticity coupling. *Phys. Rev. B* **102**, 174413 (2020).

26. S. Tateno, G. Okano, M. Matsuo, Y. Nozaki, Electrical evaluation of the alternating spin current generated via spin-vorticity coupling. *Phys. Rev. B* **102**, 104406 (2020).

27. The STAR Collaboration, Global Λ hyperon polarization in nuclear collisions. *Nature* **548**, 62–65 (2017).

28. H. Petersen, The fastest-rotating fluid. *Nature* **548**, 34–35 (2017).

29. G. Okano, M. Matsuo, Y. Ohnuma, S. Maekawa, Y. Nozaki, Nonreciprocal spin current generation in surface-oxidized copper films. *Phys. Rev. Lett.* **122**, 217701 (2019).

30. T. An, B. Cui, L. Liu, M. Zhang, F. Liu, W. Liu, J. Xie, X. Ren, R. Chu, B. Cheng, C. Jiang, J. Hu, Enhanced spin current in $Ni_{81}Fe_{19}$/Cu-CuO_x bilayer with top and sideways oxidation. *Adv. Mater.* **35**, e2207988 (2023).

31. L. Liu, T. Moriyama, D. C. Ralph, R. A. Buhrman, Spin-torque ferromagnetic resonance induced by the spin Hall effect. *Phys. Rev. Lett.* **106**, 036601 (2011).

32. S. Kasai, K. Kondou, H. Sukegawa, S. Mitani, K. Tsukagoshi, Y. Otani, Modulation of effective damping constant using spin Hall effect. *Appl. Phys. Lett.* **104**, 092408 (2014).

33. O. Mosendz, V. Vlaminck, J. E. Pearson, F. Y. Fradin, G. E. W. Bauer, S. D. Bader, A. Hoffmann, Detection and quantification of inverse spin Hall effect from spin pumping in permalloy/normal metal bilayers. *Phys. Rev. B* **82**, 214403 (2010).

34. C.-F. Pai, Y. Ou, L. H. Valelaleao, D. C. Ralph, R. A. Buhrman, Dependence of the efficiency of spin Hall torque on the transparency of Pt/ferromagnetic layer interfaces. *Phys. Rev. B* **92**, 064426 (2015).

35. S. Karimeddiny, D. C. Ralph, Resolving discrepancies in spin-torque ferromagnetic resonance measurements: Lineshape versus linewidth analyses. *Phys. Rev. Appl.* **15**, 064017 (2021).

36. T.-Y. Chen, C.-W. Peng, W.-B. Liao, C.-F. Pai, "Characterization of spin-orbit torque efficiency in the RF regime for MRAM applications," in *2021 IEEE International Symposium on Radio-Frequency Integration Technology (RFIT)* (IEEE, 2021), pp. 1–6.

37. M. Harder, Y. Gui, C. M. Hu, Electrical detection of magnetization dynamics via spin rectification effects. *Phys. Rep.* **661**, 1–59 (2016).

38. J. Sklenar, W. Zhang, M. B. Jungfleisch, H. Saglam, S. Grudichak, W. Jiang, J. E. Pearson, J. B. Ketterson, A. Hoffmann, Unidirectional spin-torque driven magnetization dynamics. *Phys. Rev. B* **95**, 224431 (2017).

39. T. Horaguchi, M. Matsuo, Y. Nozaki, Highly accurate evaluation of spin-torque efficiency by measuring in-plane angular dependence of spin-torque ferromagnetic resonance. *J. Magn. Magn. Mater.* **505**, 166727 (2020).

40. A. Hoffman, Spin Hall effects in metals. *IEEE Trans. Magn.* **49**, 5172–5193 (2013).

41. M. Matsuo, J. Ieda, K. Harii, E. Saitoh, S. Maekawa, Mechanical generation of spin current by spin-rotation coupling. *Phys. Rev. B* **87**, 180402(R) (2013).

42. D. Go, D. Jo, T. Gao, K. Ando, S. Blügel, H.-W. Lee, Y. Mokrousov, Orbital Rashba effect in a surface-oxidized Cu film. *Phys. Rev. B* **103**, L121113 (2021).

43. A. Johansson, B. Göbel, J. Henk, M. Bibes, I. Mertig, Spin and orbital Edelstein effects in a two-dimensional electron gas: Theory and application to $SrTiO_3$ interfaces. *Phys. Rev. Res.* **3**, 013275 (2021).

44. H. An, Y. Kageyama, Y. Kanno, N. Enishi, K. Ando, Spin–torque generator engineered by natural oxidation of Cu. *Nat. Commun.* **7**, 13069 (2016).

45. Y. Kageyama, Y. Tazaki, H. An, T. Harumoto, T. Gao, J. Shi, K. Ando, Spin-orbit torque manipulated by fine-tuning of oxygen-induced orbital hybridization. *Sci. Adv.* **5**, eaax4278 (2019).

46. T. Gao, A. Qaiumzadeh, H. An, A. Musha, Y. Kageyama, J. Shi, K. Ando, Intrinsic spin-orbit torque arising from the Berry curvature in a metallic-magnet/Cu-oxide interface. *Phys. Rev. Lett.* **121**, 017202 (2018).

47. J. Kim, D. Go, H. Tsai, D. Jo, K. Kondou, H.-W. Lee, Y. Otani, Nontrivial torque generation by orbital angular momentum injection in ferromagnetic-metal/Cu/Al_2O_3 trilayers. *Phys. Rev. B* **103**, L020407 (2021).

48. H. Nakayama, T. Horaguchi, C. He, H. Sukegawa, T. Ohkubo, S. Mitani, K. Yamanoi, Y. Nozaki, Spin-torque generation using a compositional gradient at the interface between titanium and tungsten thin films. *Phys. Rev. B* **107**, 174416 (2023).

49. E. Saitoh, M. Ueda, H. Miyajima, G. Tatara, Conversion of spin current into charge current at room temperature: Inverse spin-Hall effect. *Appl. Phys. Lett.* **88**, 282509 (2006).

50. H. Zhao, E. J. Loren, H. M. van Driel, L. Smirl, Coherence control of Hall charge and spin currents. *Phys. Rev. Lett.* **96**, 246601 (2006).

51. Y. Tserkovnyak, A. Brataas, G. E. W. Bauer, Enhanced Gilbert damping in thin ferromagnetic films. *Phys. Rev. Lett.* **88**, 117601 (2002).

52. H. L. Wang, C. H. Du, Y. Pu, R. Adur, P. C. Hammel, F. Y. Yang, Scaling of spin Hall angle in 3d, 4d, and 5d metals from $Y_3Fe_5O_{12}$/metal spin pumping. *Phys. Rev. Lett.* **112**, 197201 (2014).

53. K. Ando, S. Takahashi, J. Ieda, Y. Kajiwara, H. Nakayama, T. Yoshino, K. Harii, Y. Fujikawa, M. Matsuo, S. Maekawa, E. Saitoh, Inverse spin-Hall effect induced by spin pumping in metallic system. *J. Appl. Phys.* **109**, 103913 (2011).

54. E. Shikoh, K. Ando, K. Kubo, E. Saitoh, T. Shinjo, M. Shiraishi, Spin-pump-induced spin transport in p-type Si at room temperature. *Phys. Rev. Lett.* **110**, 127201 (2013).

55. P. Deorani, J. Son, K. Banerjee, N. Koirala, M. Brahlek, S. Oh, H. Yang, Observation of inverse spin Hall effect in bismuth selenide. *Phys. Rev. B* **90**, 094403 (2014).

56. Y. Wang, P. Deorani, K. Banerjee, N. Koirala, M. Brahlek, S. Oh, H. Yang, Topological surface states originated spin-orbit torques in Bi_2Se_3. *Phys. Rev. Lett.* **114**, 257202 (2015).

57. L. Zhu, R. A. Buhrman, Maximizing spin-orbit-torque efficiency of Pt/Ti multilayers: Trade-off between intrinsic spin hall conductivity and carrier lifetime. *Phys. Rev. Appl.* **12**, 051002 (2019).

58. L. Zhu, D. C. Ralph, R. A. Buhrman, Maximizing spin-orbit torque generated by the spin Hall effect of Pt. *Appl. Phys. Rev.* **8**, 031308 (2021).

59. P. M. Haney, H.-W. Lee, K.-J. Lee, A. Manchon, M. D. Stiles, Current induced torques and interfacial spin-orbit coupling: Semiclassical modeling. *Phys. Rev. B* **87**, 174411 (2013).

60. T. Seki, Y.-C. Lau, S. Iihama, K. Takanashi, Spin-orbit torque in a NiFe single layer. *Phys. Rev. B* **104**, 094430 (2021).

61. M. Aoki, E. Shigematsu, R. Ohshima, T. Shinjo, M. Shiraishi, Y. Ando, Anomalous sign inversion of spin-orbit torque in ferromagnetic/nonmagnetic bilayer systems due to self-induced spin-orbit torque. *Phys. Rev. B* **106**, 174418 (2022).

62. A. Brataas, G. E. W. Bauer, P. J. Kelly, Non-collinear magnetoelectronics. *Phys. Rep.* **427**, 157–255 (2006).
